# Hybrid Funnel Technique: A Novel Approach for Implant Site Preparation: A Pilot Study

**DOI:** 10.3390/dj10090157

**Published:** 2022-08-25

**Authors:** Luigi Canullo, Roberta Iacono, Eduardo Pires Godoy, Andrea Punzo, Alessio Cavicchia, Francesco Gianfreda, Patrizio Bollero

**Affiliations:** 1 Department of Periodontology, University of Bern, 3012 Bern, Switzerland; 2 F.A.S Screening for Prevention and Oral Health, Department of Oral and Maxillofacial Science, Sapienza University of Rome, 00185 Rome, Italy; 3 Department of Oral Biology, Faculty of Dentistry of Ribeirão Preto, University of São Paulo, São Paulo 14040-904, Brazil; 4 Independent Researcher, 00061 Rome, Italy; 5 Department of Industrial Engineering, University of Rome “Tor Vergata”, 00133 Rome, Italy; 6 Department of System Medicine, University of Rome “Tor Vergata”, 00133 Rome, Italy

**Keywords:** bone implant interactions, surgical techniques, crestal bone loss, bone condensation, bone drilling

## Abstract

(1) Background: Different techniques and tools have been developed for implant site preparation. In this clinical scenario, Hybrid Funnel Technique (HFT), a novel osteotomy procedure, has been proposed. (2) Aim: The aim of this retrospective observational study was to consider the different responses to compression of the histological bony compartments (cancellus and cortical). HFT involves the use of multiple drills for the cortical layer preparation and of an osteotome for the osteocompaction of the cancellous bone. (3) Materials and Methods: Following computer-supported implant planning and guided surgery, 10 osteotomies with HFT were performed and 10 implants with the same length and diameter were placed in seven healthy and no daily smoking patients. Periapical X-ray and intraoral photographs were performed at baseline and after 12 months of follow-up to evaluate marginal bone level (MBL) changes and aesthetic results obtained from implant prosthetic rehabilitation. (4) Results: At 1 year of follow-up, 100% of the implants were successfully integrated, MBL change mean value was 0.17 mm ± 0.21. No differences in terms of MBL were noted between thin and thick biotypes. Pink esthetic score (PES) and white esthetic score (WES), assessed one year after definitive restoration placement, were 7.5 ± 2.3 and 8.5 ± 1.1, respectively. (5) Conclusions: Based on the findings of this preliminary clinical study, HFT has led to stability of peri-implant tissues and could represent a reliable technique for surgical preparation of the implant site.

## 1. Introduction

The osseointegration of the dental implant is a delicate and complex process in which, from a biomechanical point of view, there are two different variables: primary stability and secondary stability.

Primary stability, also known as mechanical stability, is due to the mechanical friction between the implant fixture and the surrounding bone. The fundamental prerequisite to obtaining adequate primary stability is the absence of micro-movements or micro-movements between 50 µm and 150 µm [1]. Secondary stability, also known as biological stability, is linked to the healing process, and to the processes of new bone formation and bone remodeling that occur at the bone/implant interface [2].

Many factors can influence mechanical and biological stability: bone quantity and quality, osteotomy, insertion torque, implant design, and surface characteristics [3,4].

Among these, osteotomy, the surgical procedure for implant bed preparation in which the fixture is inserted, represents a factor that must be increasingly considered when planning implant-prosthetic rehabilitation. Over time, several osteotomy techniques and protocols were suggested and well described in the literature for surgical implant site preparation, and currently, there is a clear dichotomy between ablative techniques (drills and piezoelectric tips) [5,6] and expansive techniques (osteotomes) [7,8,9]. On the one hand, there is surgical preparation involving bone removal, and on the other, there is bone fragment compaction on the implant socket walls. The study of innovative techniques and technologies to improve implant site preparation is an intense research topic in dentistry [10]. 

The study and choice of the implant site preparation technique must necessarily be tailored to the site bone quality.

According to Lekholm and Zarb classification and Misch considerations, four different types of bone are distinguished from D1 to D4 [11,12].

This classification is widely used to assess site mean bone density, but in this way, the bone is considered as a single and non-stratified entity. Instead, it is necessary to consider the thickness and quality of cortical and cancellous bone. As demonstrated by Ivanova and colleagues [13], bone density affects not only primary stability but also secondary stability. 

Bone is a living tissue that responds differently to different stimuli, based on its internal structure. Many studies have shown that osteotomy influences bone response. It has been observed that a high lateral compression and high insertion torque generate mechanical stress, microfractures, necrosis, and resorption in the cortical bone [14,15].

High lateral compression and high torque curve are generated by a mismatching between implant body and implant site when the surgical site is underprepared.

Even with copious irrigation, osteotomy site preparation produces mechanical and thermal damage that culminates in a peri-implant ‘dead’ zone [16] generated during site preparation is dramatically extended when an implant is placed with a high insertion torque [17].

Toia et al. [18] observed that marginal bone level (MBL) was influenced by bone quality, implant diameter, insertion torque value (ITV), and the interaction between bone quality and ITV. It was estimated that MBL was greater with increased bone density and ITV [19].

The marginal bone resorption can remain stable over time, but it can also be the peri-implantitis trigger point.

It has been demonstrated that marrow bone responds positively to lateral compression with bone remodeling without resorption. It is richly vascularized and remodels much faster than cortical bone [14]. It can be deduced that an implant inserted with a high torque in bone type D1–D2 will show a greater MBL than an implant inserted with the same torque in bone type 4 [19].

Osseodensification has been shown to increase bone density and primary stability and secondary stability [20,21]. Therefore, osseodensification or undersized osteotomy is particularly suitable for poor density bone (D4) [22,23,24].

The need to find a balance between obtaining adequate primary stability to adopt a possible immediate loading protocol without causing damage to the peri-implant bone has led to a novel approach for the implant site preparation that combines ablative and expansive techniques. 

Although a variety of techniques are available for implant site preparation, the hypothesis of the study was that “hybrid funnel technique” (HFT), a new implant-site preparation technique, will be positively adopted to perform osteotomy, exploiting the cancellous bone elastic properties to stabilize the implant avoiding heat-friction necrosis or microfractures in the cortical bone.

The aim of the study was to introduce HFT that consists of adapting the osteotomy protocol to the type of bone in which the implant is to be inserted, preparing the two bone compartments differently. The biological rationale of the technique was to obtain a predictable primary stability in the compacted medullary bone and a better secondary stability following an accelerated intramembranous ossification in the cortical bone region. 

## 2. Materials and Methods

The present retrospective observational study was conducted during the period between September 2020 and January 2022. 

All clinical procedures were performed in accordance with the Declaration of Helsinki and the Good Clinical Practice Guidelines. Each patient enrolled in the present study signed a written informed consent form. 

The study protocol received ethical approval from the Ethics Committee of the University of Rome “Tor Vergata”, “n° 63/2022”.

Patients who required post-extraction implant-prosthetic rehabilitation in the maxillary area were enrolled and treated in accordance with the following inclusion criteria:

- ASA 1 E ASA 2;
- Age between 30 and 70 years;
- No history of periodontal disease;
- FMBS and FMPS pre-surgery <20%;
- No daily smokers.

The 10 patients underwent periodontal screening sessions of scaling and deplaquing with piezoelectric ultrasonic tips and an air polishing system. Everyone received motivation and home oral hygiene instructions regarding the correct use of toothbrushes and interdental hygiene devices. Implant surgery was performed only when full mouth plaque score (FMPS) and full mouth bleeding score (FMBS) were below the 20%. 

Intraoral scanning, photographs and CBCT images were taken for the study of clinical case and for the treatment planning to determine the correct implant placement. A surgical guide was obtained with 3D printing.

After local anesthesia (articaine plus epinephrine 1:100,000), a mucoperiosteal flap was elevated and, following the computer-guided surgery, the Hybrid Funnel Technique was performed. 

HFT consists of mimicking the shape of a funnel from a geometric point of view. In fact, the coronal part made up of the thickness of the cortex must have a larger diameter than the apical medullary bone part of the preparation. Furthermore, in the medullary part, using conical-shaped implants, it is necessary to compact the bone in a differentiated way to obtain a larger diameter in the middle third than the apex of the implant.

This technique was performed using a specially designed drill and specially designed osteotome with the implant body diameter for cancellous bone condensation. First, a 2 mm drill at implant length (10 mm) was used, subsequently, a 3–2 mm step drill and a 3.75 mm drill to remove the cortical bone were used. In the end, the osteotome with the implant body diameter was utilized for cancellous bone condensation. The osteotome (Osseotouch, Turbigo, Italy) in this circumstance was used with a hand mallet. 

After implant site preparations, dental implants were installed and tension-free soft tissue closure was achieved. Insertion torque curve and insertion torque pick value were recorded during the installation of the dental implant using a dedicated surgical handpiece (SI-1023, Implantmed Plus, W&H, Bürmoos, Austria).

No mismatching between the fixture and the peri-implant bone occurred.

All implants were immediately restored with temporary cemented (Temp Bond, Kerr, GmbH, Karlsruhe, Germany) provisional resin crowns, manufactured by dental lab. 

Sutures 6.0 (Surgiclryl-Monofast ^®^SMI, Steinerberg, Belgium) were used to adapt the flaps around the prosthetic restoration. Definitive restoration was sited after 3 months.

After surgery, a post-operative periapical radiograph was performed using portafilm Rinn XCP^®^ and repeated after 12 months.

Intraoral photos were taken during surgery, at the definitive insertion time point, and at 1 year thereafter.

Postoperative treatment included amoxycillin-clavulanic acid 875 mg +125 mg in tablet formulations twice daily for 6 days and ibuprofen 600 mg in tablet formulations twice daily for 3 days.

All patients were instructed to rinse with 0.12% chlorhexidine mouthwash for 10 days and they were on a soft and cold diet for 14 days, until suture removal. 

Moreover, patients were instructed to apply cold packs to the treated area immediately after surgery to minimize the inflammatory response and to sleep with two pillows to reduce postoperative swelling.

Patients were divided into thin and thick biotypes.

Using all the data collected, a descriptive analysis was carried out. Each variable is summarized by means of mean and standard deviation. Comparison between MBL presented in thin and thick biotypes was analyzed using T-test. Significance was set at *p* > 0.05.

The aesthetic results of the implant-prosthetic restorations were evaluated using PES and WES [25].

A clinical photograph was taken 12 months after the insertion of the final restoration, and scores were assigned for each parameter. 

## 3. Results

Individual patient characteristics and clinical measurements are summarized in Table 1. A total of seven patients were enrolled, and 10 osteotomies with Hybrid Funnel Technique were performed with 10 implants inserted. No implant failed up to 1 year after insertion, resulting in a 100% survival rate. No intraoperative and post-surgical complications were noted, in particular no patient suffered from vertigo.

The implants were placed in a bone with an average size of 640.7 HU ±145.2 corresponding to the D3 of the Mish classification [8]. The thickness of the cortical bone was 2.70 mm ± 0.86. These values were assessed on preoperative CBCT through RealGUIDE 5.0 (3Diemme, Varese, Italy).

The torque insertion curve was recorded during the implant fixture insertion directly through the implant motor. Implants were inserted with a mean torque of 28.80 Ncm ± 6.05 and the minimum value was 20 Ncm.

The difference in MBL between the two-time points (baseline and 1 year) was calculated on periapical X-rays using dedicated software. (CS 8100 3D, Carestream, Rochester, NY, USA). Bone resorption was measured on the distal and mesial side of each implant and the average between the two values was calculated to obtain MBL. Not all implants showed marginal bone changes and the MBL mean was 0.17 mm ± 0.21.

The pink esthetic score and the white esthetic score were calculated through the execution of intraoral photographs at 1 year after insertion of definitive restoration. The PES mean value was 7.5 ± 2.3, and the WES mean value was 8.5 ± 1.1.

No differences in terms of MBL were noted between thin and thick biotypes (*p* > 0.5). This section may be divided by subheadings. It should provide a concise and precise description of the experimental results, their interpretation, as well as the experimental conclusions that can be drawn.

## 4. Discussion

Various drilling techniques and tools have been tested and documented in the literature. 

The results have shown that Hybrid Funnel Technique could represent a new approach for the surgical preparation of the implant site.

This technique was born from the combination of ablative osteotomy and osteocompaction.

Conventional preparation involves the use of multiple drills of different shapes and diameters which, rotating at high speed, cut and remove bone.

Over the past decade, there has been an increasing use of piezoelectric osteotomy which typically uses vibrating tips to cut bone [26,27,28].

As demonstrated by a recent review and meta-analysis, both techniques are equal for MBL change and for the implant survival rate in the short and long term [29] (Godoy-Reina et al., 2021).

More recently, osseodensification has been proposed by Huwais and Meyer using specific burs and osteotomes which, instead of removing the bone, push it laterally, increasing the bone density of the walls of the implant site [7].

All these techniques originally do not consider the different response to compression of the histological compartments (cancellus and cortical) of the bone.

Clinicians should adopt different preparation techniques for each of them. While osseodensification appears to be the ideal technique for bone type 3 and 4, it could hinder or delay the osseointegration process in bone type 1 [30].

The HFT is performed with burs and osteotome. The drills, rotating at high speed, cut and remove the cortical bone, avoiding compression and subsequently necrosis.

In the present preliminary clinical report, 10 edentulous saddles were rehabilitated with implants with the same length and diameter inserted in a site prepared with Hybrid Funnel Technique. This technique was designed with the aim of preparing the implant site, considering the bone histology, to avoid excessive changes of the MBL. A mean marginal bone level change of 0.17 mm, slightly below traditional MBL changes after the same follow-up period, was observed after a period of 12 months [31].

If it is considered a recent review regarding MBL one year after implant placement, the results seem even more encouraging. Indeed, the review reported an MBL of 0.390 mm for immediate non-occlusal loading [32].

As regards the sum of the PES/WES values, the average results are equal to 16.00 and seem to confirm those of a recent clinical double-blind randomized study which reported, for two different types of immediate loading implants, values between 15.00 and 15.20 [33].

Therefore, the main goal seems to be achieved.

In the present study, no difference in terms of thin and thick biotypes was observed confirming the outcomes reported by the previously mentioned article.

The 10 implants were placed in the maxillary aesthetic area, and after 1 year from the insertion of the definitive restoration, the mean values of PES and WES were 7.5 mm and 8.5 mm, respectively.

As previously described, the osteotome pushed with a surgical mallet was used for compaction of cancellous bone. The use of the surgical mallet is frequently associated with intra-operative discomfort and post-operative complications such as vertigo [10].

Complications such as benign paroxysmal positional vertigo have also been observed when it is used for implant site preparation [34].

The absence of such complications and distress following the combined use of the surgical mallet and osteotome in the 10 patients treated is because the cortical bone layer had been completely removed and the osteotome was inserted into the medullary bone. The stability of the patient’s head observed during the thrusts of the surgical hammer is further proof of this assertion.

A recent review has shown that there is a relationship between implant macro-geometry and the size of the osteotomy that can influence the osseointegration process [19].

According to Coelho and Jimbo [35], the type of osteotomy can lead to a remodeling of the interfacial bone. This assumption clinically implies that when there is bone compression, the osseointegration process is slowed down by the need to reabsorb the tissue necrosis before the apposition of new bone [36]. In contrast, by inserting implants that had a diameter equivalent to the osteotomy, the formation of membranous-like bone healing chambers was observed [37]. In fact, in these areas, the space between the implant and the osteotomy allows faster bone healing since it is not necessary to reabsorb the necrotic bone [38]. This phenomenon could compensate for the loss of primary stability of the implant over time to the advantage of faster secondary stability in the proximity of the area where ablation of the cortical segment was performed [39].

The HFT might suggest that segmented osteotomy can promote primary stability in the apical part where osseodensification was performed. At the same time, in the cortical osteotomy area, the more rapid bone formation through the healing chambers for intramembranous ossification can promote faster secondary stability. Assessment of implant stability by resonance frequency analysis with the recording of the ISQ values at the time of implant placement and also at pre-established timepoints, could provide more information to support HFT and time of osteointegration [40,41,42]. Although it is important to remember that the ISQ value must be interpreted with caution as it does not intrinsically represent an index of the quality of osseointegration [40]. As the present is a preliminary study, the main limitations of the present study were represented by the short follow-up period, the small sample size, and the fact that the implant stability over time was not considered.

Another limit to be explored can be the placement of implants in the intra-foramina region. Indeed, the axis of the osteotomy can in the apical portion of the osteotomy involve the lingual cortex making it difficult to segment the zone between bone ablation and bone compaction.

Authors should discuss the results and how they can be interpreted from the perspective of previous studies and of the working hypotheses. The findings and their implications should be discussed in the broadest context possible. Future research directions may also be highlighted.

## 5. Conclusions

However, the study may represent a preliminary report on the potentiality of the new preparation technique exploiting peculiar features of drilling and osteocondensation.

Despite the limitations of the present study, preliminary data show that the funnel technique leads to stability of the peri-implant tissues, successfully integrated implants, and satisfactory aesthetic results.

Future studies will need to evaluate long-term data from applying the technique to different bone densities.

## Figures and Tables

**Table 1:** Patient characteristics and main results.

	Age	Sex	Bone Density (HU)	Cortical Thickness (mm)	ITV (Ncm)	MBL (mm)	PES	WES
Mean	41.3	58% Female; 42% Male	640.7	2.70	28.80	0.17	7.5	8.5
SD	5.5	-	145.2	0.86	6.05	0.21	2.3	1.1

## Data Availability

Not applicable.
